# It’s What We Do: Experiences of UK Nurses Working during the COVID-19 Pandemic: Impact on Practice, Identity and Resilience

**DOI:** 10.3390/healthcare10091674

**Published:** 2022-09-01

**Authors:** Zoe Davey, Cynthia Srikesavan, Andrea Cipriani, Catherine Henshall

**Affiliations:** 1 Oxford Institute of Nursing Midwifery and Allied Health Research, Faculty of Health and Life Sciences, Oxford Brookes University, Oxford OX3 0FL, UK; 2 Oxford Health NHS Foundation Trust, Warneford Hospital, Oxford OX3 7JX, UK; 3 Department of Psychiatry, University of Oxford, Oxford OX3 7JX, UK; 4 Oxford Precision Psychiatry Lab, NIHR Oxford Health, Oxford OX3 7JX, UK

**Keywords:** resilience, mental health, burnout, COVID-19, nursing, focus groups

## Abstract

The COVID-19 pandemic increased pressure on a nursing workforce already facing high levels of stress, burnout, and fatigue in the United Kingdom (UK) and internationally. The contribution of nurses to keeping the public safe was widely recognised as they met the challenges of delivering complex patient care during the healthcare crisis. However, the psychological impact of this on nurses’ health and wellbeing has been substantial, and the number of nurses leaving the profession in the UK is rising. The aim of this study was to explore the experiences of nurses working during the COVID-19 pandemic and the impact of this on their psychological health, wellbeing and resilience. The study is part of a wider project to develop and pilot an online resilience intervention for nurses during COVID-19. Five focus groups with 22 nurses were carried out online. Data was analysed thematically using the Framework Method. Four key themes relating to positive and negative impacts of working during the pandemic were identified: Rapid changes and contexts in flux; loss and disruption; finding opportunities and positive transformation; and reinforcing and strengthening identity. Implications for coping and resilience in nursing, nursing identities and workforce development are discussed.

## 1. Introduction

The COVID-19 pandemic has been an unprecedented challenge for health and care systems globally, with significant implications for the workforce that are likely to be felt for some time to come [1,2]. Frontline healthcare professionals worked tirelessly to deliver expert patient care, whilst also overseeing wide ranging challenges including staff sickness, personal protective equipment requirements, rapidly evolving clinical care policies and previously unseen patient care demands [3,4]. The first positive cases of COVID-19 were identified in the United Kingdom (UK) in January 2020; by March 2020 the UK was placed in lockdown accompanied by a range of policies and restrictions, including social distancing, the closure of schools and businesses, and stay at home measures, which were aimed at reducing transmission of the virus and minimizing the impact on the health service [5]. Following this first lockdown, the government response to subsequent waves of the pandemic throughout 2020 and 2021 included the tightening and easing of these restrictions, and the introduction of local and national lockdowns alongside the rollout of a national vaccination programme.

The pandemic has taken a substantial personal toll on healthcare professionals globally, with the World Health Organisation (WHO) estimating between 80,000 and 180,000 deaths from COVID-19 in this professional group between January 2020 and May 2021 [6,7]. Moreover, evidence suggests that the psychological impact of working in these intense clinical settings has been considerable, and healthcare professionals working during the pandemic have reported increased levels of stress, distress, anxiety, and depression [8,9,10].

The contribution of nurses, in particular, to keeping the public safe during COVID-19 has been widely recognised, helping to reinforce images of nurses as heroes and angels that have proliferated the public discourse for decades [11]. However, the pandemic emerged at a time when the nursing workforce in the UK was already under significant strain, with increased burnout, reduced job satisfaction, and problems of recruitment and retention fuelled by staff and infrastructure shortages, inadequate pay structures and opportunities for career progression, racial inequalities, and chronic, excessive workloads [12,13]. The pandemic further exacerbated existing workforce supply issues, and the number of nurses leaving the profession in the UK has started to rise [14].

Personal resilience has been highlighted as an important tool for nurses and other healthcare professionals to cope with adverse workplace experiences [15,16,17]. The protective role of resilience has been identified as important for nurses learning to manage the ongoing pressures of working during COVID-19 [18,19,20,21]. However, the term resilience has been contested within the literature, with resilience being conceptualized as both a personality trait and an adaptive and dynamic process [22]. The current study draws on the concept of personal resilience as the ability to ‘cope successfully despite adverse circumstances’ [23]. This acknowledges that whilst most nurses have a baseline resilience to cope with daily workplace stressors, the daily challenges encountered by nurses can impact their ability to maintain that resilience.

Wellbeing is a related concept that is often difficult to define, but is similarly associated with an individual’s ability to deal with the complex, varied and multi-dimensional stressors encountered in the workplace and in daily life [24]. Positive psychological health and mental wellbeing in particular are associated with an individual’s ability to cope with stressors, function effectively, and develop positive relationships [25]. The importance of the wellbeing of healthcare professionals is being increasingly highlighted within the literature, and is closely linked to concepts of resilience and burnout [24].

Providing support to enhance and strengthen personal resilience can promote psychological health and wellbeing, and improve recruitment and retention [26,27]. Similarly, the development of evidenced-based strategies to improve the psychological wellbeing of healthcare staff and mitigate future burnout following the COVID-19 pandemic has been emphasized as a key priority [28]. This is in line with the UK National Health Service (NHS) Health and Wellbeing Framework, which advocates for the implementation of staff health and wellbeing plans to support staff to feel well, healthy, and happy at work [29]. To develop tailored and relevant support programmes for UK nurses, an understanding of the impact of the COVID-19 pandemic on nurses, both personally and professionally, is required. Whilst studies have examined the experiences of different healthcare professionals working in the UK during COVID-19, few have examined the experiences of nurses specifically [30,31,32,33]. Moreover, further work is required to examine the impact of COVID-19 on nurses’ resilience and to explore their experiences of, and access to, support.

## 2. Materials and Methods

The aim of the current study was to explore the impact of working during COVID-19 on the resilience, psychological health, and wellbeing of nurses.

### 2.1. Study Design

This qualitative focus group study was part of a larger mixed methods project to develop and pilot an online resilience training programme for nurses (REsOluTioN) [34]. A qualitative approach was adopted for this phase of the study in order to generate rich data to help develop a better understanding of nurses’ experiences [35]. Focus groups are effective within health services research for examining people’s knowledge and experiences, using group dynamics to explore and clarify views and perspectives, and allowing participants to discuss issues of importance to them [36]. Focus groups have also been found to be useful in studies involving healthcare professionals [36]. Focus groups in the current study were carried out online so that they could take place in the context of COVID-19 related workplace restrictions and risk assessments. However, the use of video conferencing platforms for focus group studies has also been found to be a particularly cost-effective method for engaging geographically spread, time-poor participants [37]. The focus group study took place at an NHS Trust in the South of England from August to September 2021, during the height of the pandemic. The Trust is a community and mental health trust that provides physical, mental health, and social care services to children and adults.

### 2.2. Sampling, Access and Recruitment

Nurses were recruited opportunistically through Trust communications and newsletters, posters, and social media. Nurses from different fields of nursing, working in a range of clinical settings (e.g., adult, mental health, children and young people, community and district nursing, health visiting, and management) and from a range of clinical bands were recruited to take part.

### 2.3. Patient and Public Involvement

In line with other studies focusing on healthcare staff as participants, the current study engaged role-holder PPI participants [38]. Nurses provided valuable input and feedback throughout the study to ensure that their views were incorporated into the study design and process from the outset. Nurses were also involved in the wider REsOluTioN project [34], to inform the development of the resilience training programme and recommendations arising from study findings.

### 2.4. Data Collection and Analysis

Nurses who expressed an interest in participating were provided with a link to an online information sheet and consent form on the Qualtrics survey platform. Focus groups were carried out online via the Zoom platform, lasted approximately 1 h and were facilitated by two members of the research team who were experienced in facilitating focus groups with healthcare professionals. Clear ground rules were established at the outset of the focus groups in order to promote an open discussion, encourage contributions from all participants and maintain confidentiality. The focus group topic guide explored how working as a nurse during the COVID-19 pandemic impacted participants’ levels of resilience and overall wellbeing personally and professionally, the extent to which the pandemic impacted on patient care, relationships with colleagues and their work as nurses, and the support provided to and accessed by nurses. Given the potentially sensitive nature of the topics and experiences discussed, participants were also provided with details of further support that could be accessed outside of the focus group and their right to withdraw from the study at any time without giving a reason was emphasized. Focus groups were recorded online and transcribed by a local transcription company. Transcripts were de-identified and data were analysed thematically. All transcripts were coded by a member of the research team. The Framework Method was used to organise and chart data to allow emerging themes to be compared and contrasted across and within cases [39]. Themes were discussed and agreed by the research team at regular study meetings.

The study was approved by the Oxford Brookes University research ethics committee (F.20.01.12), the NHS Health Research Authority (21/HRA/1418), and the participating trust’s research and development department. Informed consent was obtained from all participants involved in the study prior to the focus groups commencing.

## 3. Results

Twenty-two registered nurses who had worked during the COVID-19 pandemic were recruited to take part in one of five focus groups. Demographics of focus group participants are presented in Table 1.

Four interrelated themes related to the impact of working during COVID-19 were identified from the focus group data: ‘Rapid changes and contexts in flux’; ‘loss and disruption’; ‘finding opportunities and positive transformation’; and ‘reinforcing and strengthening identity’. These themes are discussed below.

### 3.1. Rapid Changes and Contexts in Flux

#### 3.1.1. Clinical Practice, Settings and Patient Care

Participants highlighted significant changes to their clinical settings, tasks and duties, interactions with patients, and day to day work during the COVID-19 pandemic. Changes were closely related to field of nursing, clinical environment, and the specific needs of their patient groups.

*“It changed drastically...everybody was working from home...You only saw a client face-to-face in an emergency...We had a duty system where there was just two people in the office, admin worked from home...And also, our case loads went up”* (FG4, Female, Band 6, Community Mental Health).

Despite these differences, there was a sense from all participants that these workplace changes were profound and far reaching. Moreover, changes were rapid and dynamic, as the Trust continually implemented new policies and procedures in response to changes in government guidance, rates of infection, and growing evidence around transmission and treatment of the virus.

*“I don’t want to keep using the word challenging, because it’s been profound. I think it’s been profound in nursing, for healthcare generally…It’s been, by far, the hardest 20 months of my nursing career. And I’ve been a nurse for a long time”* (FG5, Female, Band 8, Management).

Despite this, many participants reported that some systems and processes put in place during this period were likely to remain post pandemic, particularly around infection control, risk management, and the delivery of patient care.

*“And also, for us, protecting our older people. I think we’re going to be in masks forever. I can’t ever see us stopping now”.* (FG4, Female, Band 6, District Nursing).

#### 3.1.2. Staffing and Job Roles

Participants emphasised the challenges associated with the impact of COVID-19 on staffing and job roles. Changes to service structures and delivery during the pandemic resulted in many staff being redeployed into different frontline clinical areas. Whilst the bolstering of staff numbers through the redeployment of nurses into unfamiliar contexts and environments helped to overcome some of the challenges associated with staff absences, it also placed substantial strain on existing staff members who had to provide training and support to redeployed staff, leading to role burden and confusion.

*“We have been the recipients of loads of redeployed staff, which was very helpful, from other services to support us. But that has created a big impact on our own staff…to support redeployed staff coming from other areas, very willing and very capable, but not familiar with our environment”* (FG1, Female, Band 8, Community).

#### 3.1.3. Wider Social Change

In addition to the rapid flux within clinical settings, participants also referred to the impact of wider contextual changes, such as managing the large-scale societal upheaval taking place in the UK during COVID-19. This led to substantial changes in participants’ lives, particularly in the earlier stages of the pandemic when strict restrictions were imposed on social interactions, travel, and business. Participants also reported making huge sacrifices in their personal circumstances in order to keep friends and family safe whilst they were working, including changing their living arrangements, making changes to physical spaces and processes at home and limiting contact with family and friends. However, as the pandemic progressed and restrictions were eased and the public started to return to pre-pandemic behaviour, participants’ work lives were often at odds with their home lives.

*“My children went to go live with their dad for three months while I [was redeployed]. Because, obviously, we didn’t know what was going on at that time. We didn’t know how scary the disease was. We had no concept. And my daughter has asthma, so I was being super careful. It was a very weird and frightening time…It’s strange now when people talk about the beginning of the pandemic and they’re all talking about making sourdough bread and watching Tiger King. And I just think, no, actually, it was really horrible”* (FG2, Female, Band 7, Management).

### 3.2. Loss and Disruption

#### 3.2.1. Resilience and Wellbeing

Working during the COVID-19 pandemic placed significant strain on participants’ mental and physical health, leading to exhaustion, testing their resilience, increasing levels of anxiety and negatively impacting on wellbeing. Participants also expressed loss of self-efficacy, certainty, confidence, and control in their ability to adapt to changing circumstances, deliver the best quality patient care, and look after their colleagues, friends and family.

*“Trying to balance everything has been incredibly difficult…I’m beyond tired. My resilience has really been tested. And it’ll take a while to come back from that…there were times I worked 35 days without a day off…There wasn’t anybody else to do it, because of the team size, and because of the demand of the service. And so that absolutely has to have a knock on physical and mental impact”* (FG5, Female, Band 8, Management).

*“It was just exceptionally stressful, because we didn’t feel like we could nurse as we can nurse, as we were taught to nurse, and how it is in us to nurse. And then obviously we all had the fear of taking COVID back home with us”.* (FG3, Female, Band 6, Community).

#### 3.2.2. Support Structures and Coping Mechanisms

The pandemic also disrupted many of the various support structures and coping mechanisms traditionally used by participants to manage work-related stress. Most significantly, this included the dislocation of team working, leading to less frequent handovers, debriefs and incidental conversations, fewer opportunities to share experiences, and increased isolation.

*“I was just suddenly very conscious of being on my own and just having to get on with it…And that all those the corridor chats there, you lost all that support didn’t you, that talking with colleagues and saying, I’m not coping with this or I am…That, just overnight disappeared”* (FG4, Male, Band 8, Learning Disabilities).

In addition, participants highlighted the disruption of family and peer support networks, the suspension of various health and wellbeing activities outside work, and the loss of work–life balance as having hindered their ability to manage and recover from the impact of working during the pandemic.

*“To be a nurse, you need to have somebody to offload to, to support you, no matter what job you do in health care, whether it’s your team that support you or at home. And if you haven’t got that that’s quite hard, and I think we didn’t all have that for a little while”* (FG5, Female, Band 5, Community).

*“You couldn’t get away from it. That first lockdown, you couldn’t get away from it”* (FG3, Female, Band 8, Management).

### 3.3. Finding Opportunities and Positive Transformation

#### 3.3.1. Networks and Teams

Despite substantial disruptions, participants did identify opportunities to develop new networks and priorities. Many participants commented on the extent to which working during the pandemic had brought teams together and helped to forge new networks of support.

*“We have a lot of camaraderie now between community hospitals that we didn’t have before, because we’ve had to share staff, we’ve had to share equipment. But I’d like to think that that camaraderie will carry on”* (FG3, Female, Band 6, Community).

#### 3.3.2. Organisational Support

Furthermore, participants spoke positively about how the pandemic had helped to bring the mental health and wellbeing of staff to the forefront of NHS organisations. The Trust introduced a range of policies and programmes of support for staff to access and some participants suggested that a more sensitive and aware cultural shift had taken place.

*“In some ways I think, if anything, this is probably heightened to senior management that we need to look after our staff mentally. Where before it was always a subject…I think in some ways it got forgotten about. And during this period that’s highlighted that even more. So, I think the stigma on lots of mental health has hopefully maybe been addressed to some extent. And there are lots of services that are offered for our nursing staff, which is great”* (FG5, Female, Band 7, Community).

However, participants did highlight barriers to accessing the support that was in place, including increased workload and a lack of time. Moreover, some participants reported a withdrawal of some of the pastoral support put in place during the acute stages of the pandemic, in line with an expectation of getting back to normal.

*“I’m sure there probably was everything we needed out there, but then it was just finding the time to access what was out there, because every shift was busy. Every shift was stressful. Everyone said from the beginning, you are last. You put yourself last. So, certainly, probably, from my point of view, I didn’t make enough time to access what I needed”* (FG3, Female, Band 5, Mental Health).

*“I found that we had all this staff right at the beginning, but when our resilience had run out, and we were [exhausted], and we were fed up, they disappeared”* (FG4, Male, Band 6, District Nursing).

### 3.4. Reinforcing and Strengthening Identity

#### 3.4.1. Nursing Identity as Support

The COVID-19 pandemic had a complex impact on many participants’ nursing identities. On the one hand, feelings of duty and the need to ‘do something’ were sources of motivation for participants. Participants reported feeling immense pride at being part of the nursing workforce during this period and reported that working during the pandemic helped them to shape and reconfirm their own senses of identity as nurses. Participants reflected on how visible support from the community such as the ‘Clap for Carers’ campaign and the delivery of support and care packages from different community organisations helped to strengthen their resolve to continue.

*“I think we should be proud of ourselves in how we’ve overcome this as nurses. And as teams, as well, within the Trust, and wider NHS services, because at the end of the day we’re all still here, and we’re doing our job to our best capacity. And in some ways, I’m really glad of everyone for doing that”* (FG5, Female, Band 7, Community).

For many, working during the COVID-19 pandemic was characterised by a sense of pushing through any difficulties and getting on with job, because that is what being a nurse was perceived to entail. However, living up to this view of nurses was not always reported to be beneficial for participants. Participants gave examples of nurses quickly adapting to new modes of practice and of going above and beyond to deliver best patient care under extreme circumstances, but also of working to the point of exhaustion and physical illness. Participants also identified a perceived expectation from the Trust to return to ‘normality’ as quickly as possible, even if this meant not allowing themselves the time to properly process their experiences and recover from the strains they were under.

*“We just do carry on…You had a job to do and that was it. People were clapping for you every night. You couldn’t let these people down. People rely on you. If you didn’t go, no one would go”* (FG4, Male, Band 6, District Nursing).

#### 3.4.2. Re-Evaluating and Reshaping Nursing Identity

Conversely, others found that working during COVD-19 led them to re-evaluate and challenge some of the assumptions previously held about nursing as a profession, particularly around psychological wellbeing and safer working practices. Participants felt that they were able to prioritise their own mental health and wellbeing more at work, as well as being able to ask managers within their own trusts for greater assistance and resources to support this.

*“There was a big thing…this year where they turned around and said, stop calling NHS workers heroes. Because if you put the title of hero to someone, you stop asking if they’re okay and if they can cope”* (FG4, Male, Band 6, District Nursing).

## 4. Discussion

The primary aim of this focus group study was to explore the impact of working during COVID-19 on the resilience, psychological health, and wellbeing of nurses. The study also examined nurses’ experiences of and access to support during this period.

### 4.1. Rapid Changes and Contexts in Flux

The findings indicate that working during the COVID-19 pandemic was characterised by rapid and significant contextual change. Clinical practice, nursing settings, and job roles were all adapted in line with new policies and procedures adopted to control the spread and minimize the risk of COVID-19 to patients and staff. Previous research indicates that organisational change can lead to psychological stress, symptoms of burnout, and loss of professional efficacy in nurses even in non-pandemic times [40]. Moreover, burnout and secondary traumatic stress experienced in non-crisis periods have been found to be associated with increased anxiety and depression in nurses [41].

However, nurses’ experiences of working during the pandemic not only impacted their work lives, but also had a profound impact on their home lives. Participants highlighted the challenges associated with managing both the effects of the pandemic on wider society as well as the stress associated with working in a higher risk workplace. Fear of infection and fear of passing on infection to family and friends was a source of stress and anxiety for nurses, leading many to isolate from family friends for prolonged periods [42]. This persistent, global state of flux experienced by nurses may further contribute to burnout, as well as putting them at increased risk of compassion fatigue [43]. Stevenson and colleagues [44] found that nurses experiencing compassion fatigue as a result of increased workplace stressors and adverse events encountered during the COVID-19 pandemic, also experienced greater parental burnout, child abuse, child neglect, spouse conflict, and substance abuse.

### 4.2. Loss and Disruption

Participants in the current study reported exhaustion and physical illness, depleted resilience and wellbeing, and increased experiences of anxiety, depression, stress and trauma as a result of working during the COVID-19 pandemic. This is consistent with previous research examining the psychological impact of working during the pandemic on other healthcare professionals [45,46] and paints a picture of a workforce under significant and persistent strain.

Findings also confirmed the extent to which nurses currently rely on peer support (e.g., staff handovers, staffroom and other incidental conversations) and informal non-workplace support networks and coping mechanisms (e.g., family, friends, exercise, hobbies), to manage workplaces stress and adverse events [47]. However, the disruptive nature of COVID-19 meant that many of these support networks and mechanisms were no longer available and the move to homeworking combined with the pervasive impact of the pandemic on wider society meant that nurses were unable to maintain work–life balance and separation as a way of coping with multiple challenges. The medium and long-term consequences of the multiple sources of stress and distress encountered by frontline healthcare workers including nurses during COVID-19 are not yet known. Research looking at other infectious diseases (e.g., Ebola, SARS, MERS) suggest that the behavioural and psychological consequences can last months and even years after the acute phase of these outbreaks [48,49].

### 4.3. Finding Opportunities and Positive Transformation

Developing and implementing evidenced-based programmes of support targeted at improving wellbeing and building resilience has been identified as a key priority in the COVID-19 recovery process [1]. Throughout the pandemic the NHS has continued to prioritise a coordinated approach to supporting staff, placing a clear focus on the health and wellbeing of the workforce [50]. The importance of good nursing leadership and effective team dynamics in supporting the nursing workforce, and the impact of this on nurses’ wellbeing and the delivery of nursing care has been highlighted [51]. The COVID-19 pandemic has introduced additional challenges for clinical teams, particularly in the face of fluctuating staff levels and redeployment, changing patient needs, and a constantly evolving clinical picture [52]. Findings from the current study did indicate an uplift in the provision of organisational support during the pandemic, including wellbeing sessions, supervision, and care packages. Participants in the current study also highlighted the importance of good leadership, teamworking, and building strong relationships on care quality and staff wellbeing. However, barriers to accessing support were reported, the most prominent of which appeared to be time. Moreover, the experience of nurses working during COVID-19 did uncover some of the gaps in structured organisational support available to them, particularly in the absence of the frequently relied upon support structures outside of the workplace.

### 4.4. Reinforcing and Strengthening Identity

Successful system level change in how nurses are supported and how staff mental health and wellbeing are prioritised requires not only a structural shift but also a cultural shift, including reshaping nurse identities. Study findings indicate that the perception of nurses as ‘heroes’ or ‘angels’, and the sense of duty towards patients and others that stems from this, could be a positive source of strength and motivation. However, it has been argued that the angel/hero construct has a number of unintended consequences and ultimately undermines the nursing profession [10]. Findings from the current study also suggests that this ‘superhuman’ identity does not encourage nurses to prioritise their own mental health and wellbeing and is therefore unsustainable. Reframing the nursing identity to include an understanding of resilience as a crucial but dynamic process requiring continuous nurturing and commitment that still engenders strength and pride, but also encourages help seeking, reflective practice and self-care, would have positive implications for individuals and for the nursing workforce as a whole [53].

The extent to which strengthening resilience can also be understood as an organisational responsibility, as part of a collective approach to supporting wellbeing and positive psychological health, should also be considered [54]. Findings from a recent scoping review suggests that different interventions aimed at building and maintaining the resilience of frontline healthcare professionals during COVID-19 are required at individual, organisational and environmental levels [55].

### 4.5. Policy and Practice Implications

Findings from the current study further highlight the need for tailored, evidence-based interventions for nurses across healthcare settings to strengthen resilience and maintain positive psychological health and wellbeing. The need for structured support to be embedded into nurses’ job roles, to provide mentorship and advice, solve problems, and provide time out of the clinical environment is emphasized. At an organisational level, resilience interventions need to be integrated into local and national healthcare service policies and be included as a central component of workforce development plans. However, the provision of effective and targeted education, training, and interventions to nurses and nurse managers is not sufficient for protecting the mental and physical health, resilience, and wellbeing of the nursing workforce. Investment in psychosocial support, monitoring, and treatment is also required alongside carefully considered, well-defined job roles and responsibilities, healthy work patterns and facilitative working conditions [55].

### 4.6. Limitations

Restriction of the current study to a single NHS Trust in the South of England may be a limitation for the transferability of findings. Moreover, the study was relatively narrow with regard to ethnicity. Whilst participants from a range of nursing fields and clinical settings were recruited to participate in the focus groups, and their experiences are in line with evidence from research in other healthcare professions and regions, future studies should seek to recruit nurses from across a wider geographic area and with an emphasis on inclusion and diversity. Disparities with regard to experiences, risk of exposure and infection, access to PPE, testing and vaccination, and physical and mental health outcomes among different ethnic groups and across different regions have been identified [56,57,58,59].

## 5. Conclusions

The COVID-19 pandemic placed additional strain on an already overstretched nursing workforce and prioritising the mental health and wellbeing of nurses will be an important component of the recovery process. Proactively addressing nurses’ needs through the development and implementation of integrated support programmes and pathways, including those which specifically target resilience, could help to develop more realistic and sustainable nursing identities, with substantial implications for nursing recruitment and retention and wider benefits for service delivery and patient care.

## Figures and Tables

**Table 1:** Participant demographics.

Demographic		N (%)
Age	18–29	1 (4.55)
	30–39	4 (18.18)
	40–49	8 (36.36)
	50–59	7 (31.82)
	60+	2 (9.09)
Gender	Female	19 (86.36)
	Male	3 (13.64)
Band	5	4 (18.18)
	6	6 (27.27)
	7	6 (27.27)
	8	5 (22.73)
	Other	1 (4.55)
Ethnicity	White British/Other White Groups	21 (95.45)
	Other	1 (4.55)
Years working in profession (years)	1–5	3 (13.64)
	6–10	4 (18.18)
	11–15	3 (13.64)
	>15	12 (54.55)

## Data Availability

The data presented in this study are available on request from the corresponding author.
